# Enhancing visualization with low echo reduction during endoscopic ultrasound-guided pancreatic duct drainage

**DOI:** 10.1055/a-2239-3589

**Published:** 2024-01-30

**Authors:** Haruka Toyonaga, Tsuyoshi Hayashi, Masayo Motoya, Toshifumi Kin, Kuniyuki Takahashi, Akio Katanuma

**Affiliations:** 1 Center for Gastroenterology, Teine Keijinkai Hospital, Sapporo, Japan

Endoscopic ultrasound-guided pancreatic duct drainage (EUS-PD) is performed as an alternative when conventional endoscopic pancreatic duct drainage has failed 1 . Although accurately puncturing a narrow pancreatic duct remains challenging, contrast-enhanced EUS has been reported to be effective in puncturing poorly visible ducts 2 3 . Low echo reduction (LER) mode, which is incorporated into a new EUS processor (EVIS EUS EU-ME3; Olympus, Tokyo, Japan), enhances visibility by suppressing low echo signals while maintaining high echo areas 4 5 ; this method may improve EUS-PD outcomes without using contrast media.

In a challenging case of pancreatitis due to pancreaticojejunal anastomotic stenosis after subtotal stomach-preserving pancreaticoduodenectomy ( Fig. 1 ), balloon enteroscopy-assisted endoscopic retrograde cholangiopancreatography was unsuccessful due to postoperative adhesions. Hence, EUS-PD was performed using the echoendoscope UCT-260 (Olympus) and EUS processor EU-ME3. To ensure sufficient working space and stent placement distance, we attempted to puncture the upper stream of the pancreatic duct. With a pancreatic duct diameter of just 1.2 mm and poor visibility, puncturing proved challenging. By employing LER, the duct was depicted as a lower echo structure, emphasizing the echo brightness difference with the pancreatic parenchyma, improving visualization ( Fig. 2 , Fig. 3 **a** ). Successful puncture of the pancreatic duct was achieved using a 22-gauge needle. The nearly perpendicular intersection of the puncture needle and the pancreatic duct posed a challenge in advancing the guidewire ( Fig. 3 **b** ). After re-evaluating the puncture location ( Fig. 4 ), a spot closer to the anastomosis was punctured, confirmed with contrast, and the guidewire was advanced. After substituting the needle for a catheter and dilating the puncture route ( Fig. 5 ), a 7 Fr × 14 cm plastic stent was placed from the stomach through the pancreatic duct to the jejunum.

**Fig. 1:**
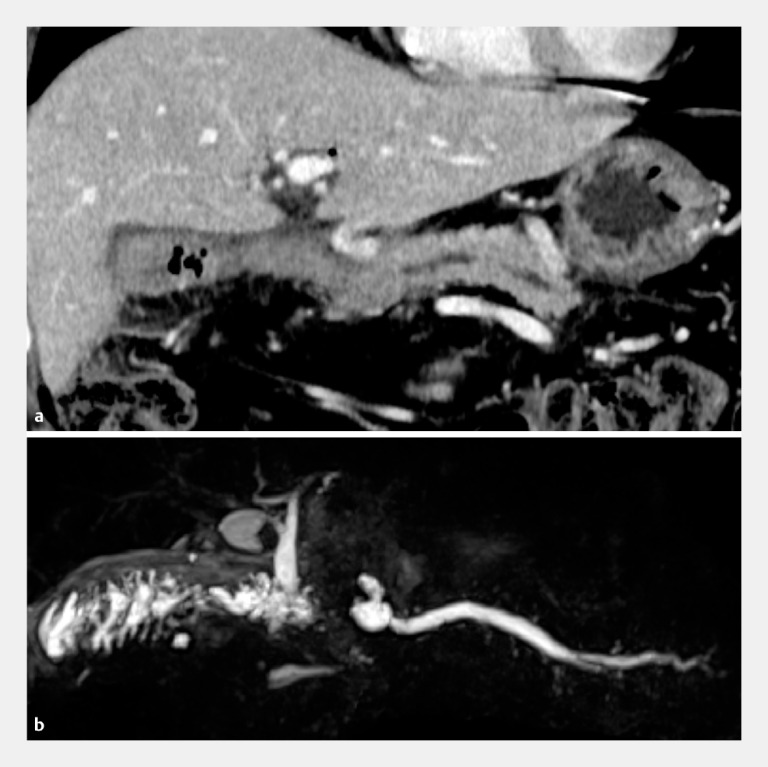
Imaging studies of a patient who suffered from pancreatitis due to pancreaticojejunal anastomotic stenosis. **a** Contrast-enhanced computed tomography. **b** Magnetic resonance cholangiopancreatography.

**Fig. 2:**
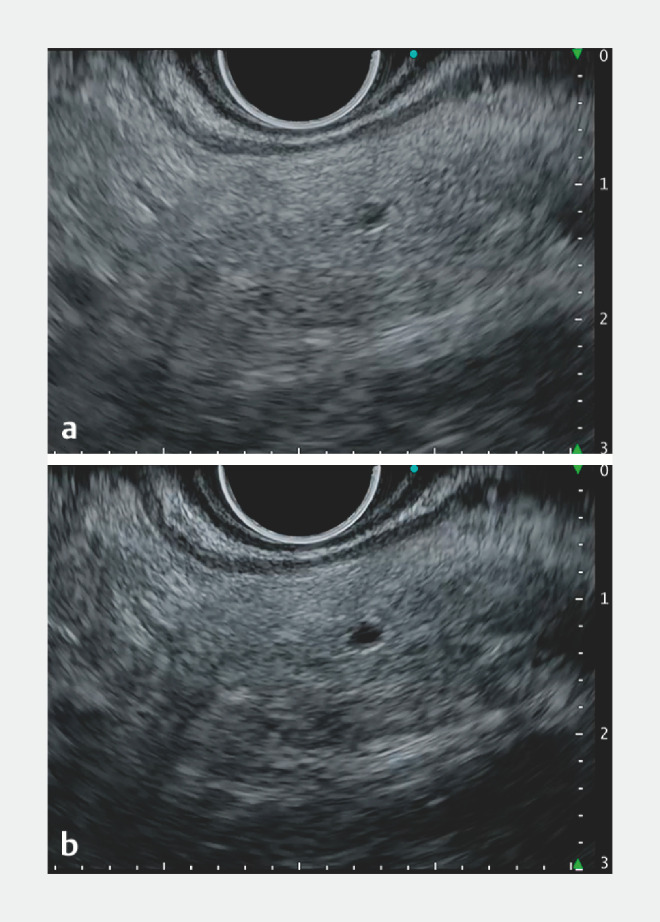
Endoscopic ultrasound (EUS) images of the upper stream of the narrow pancreatic duct (1.2 mm in diameter) during EUS-guided pancreatic duct drainage. Low echo reduction (LER) enhances the contrast by suppressing the low echo areas without overemphasizing high echo areas. The LER ranges from Lv.1 to 20 and is usually set at Lv.3. **a** LER Lv.3 (default). **b** LER Lv.7 (enhancing).

**Fig. 3:**
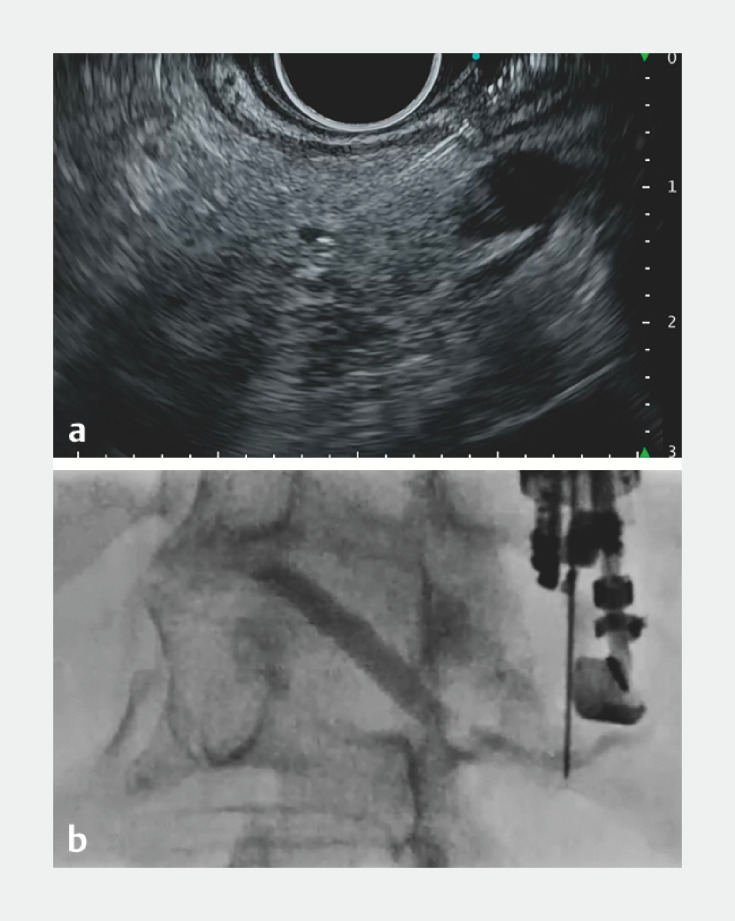
Puncture of the pancreatic duct. **a** Endoscopic ultrasound image: low echo reduction enhanced visibility of the narrow pancreatic duct, which the needle then successfully punctured. **b** Fluoroscopic image: the nearly perpendicular intersection of the puncture needle and the pancreatic duct posed a challenge in advancing the guidewire.

**Fig. 4:**
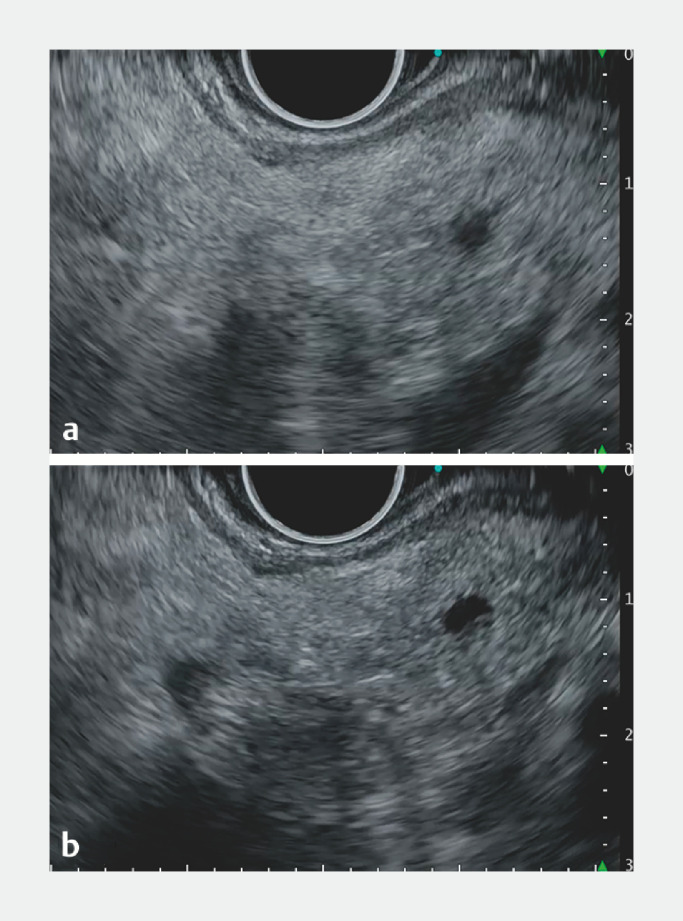
Endoscopic ultrasound (EUS) images of the main pancreatic duct (1.8 mm in diameter) closer to the anastomosis during EUS-guided pancreatography drainage. **a** Low echo reduction (LER) Lv.3 (default). **b** LER Lv.7 (enhancing). LER enhanced the contrast without overexposure, and improved visibility of the pancreatic duct.

**Fig. 5:**
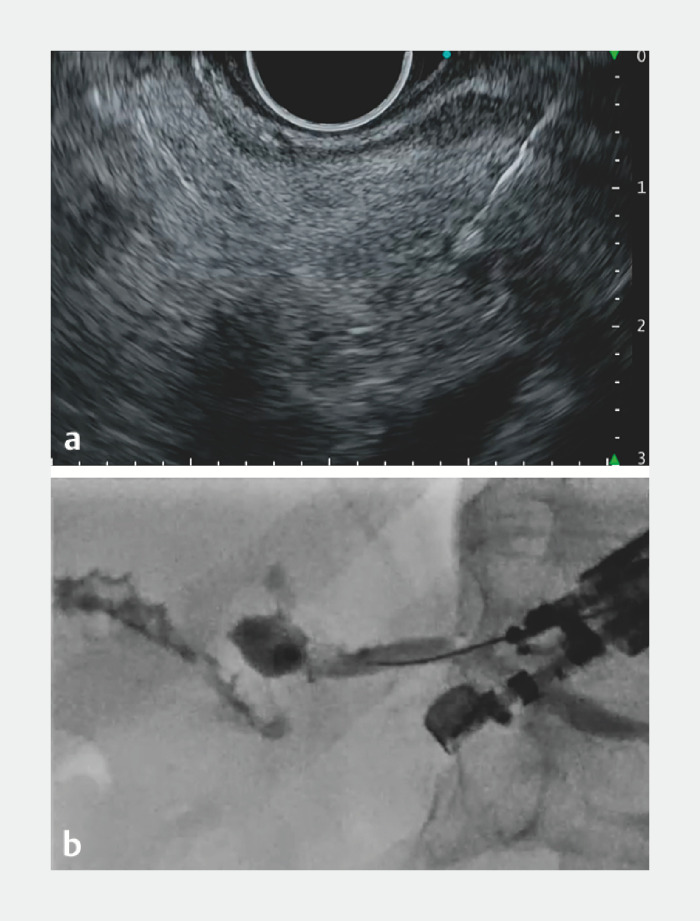
Successful puncture of the pancreatic duct, contrast of the pancreatic duct and afferent limb, and advancement of the guidewire. Finally, a 7 Fr × 14 cm plastic stent was placed from the stomach through the pancreatic duct to the jejunum after dilation of the puncture route. **a** Endoscopic ultrasound image. **b** Fluoroscopic image.

Despite advances in interventional EUS, EUS-PD remains a complex procedure with a high complication rate. LER is a promising image-adjustment feature that may improve visibility and puncture success rates in EUS-PD ( Video 1 ).

Novel low echo reduction modality incorporated into a new endoscopic ultrasound (EUS) processor (EU-ME3; Olympus, Tokyo, Japan), which suppresses low echo areas while maintaining high echo areas to improve the visibility of narrow pancreatic ducts during EUS-guided pancreatic duct drainage.Video 1

Endoscopy_UCTN_Code_TTT_1AS_2AD

## Notes

Endoscopy E-Videos https://eref.thieme.de/e-videos
*E-Videos* is an open access online section of the journal *Endoscopy* , reporting on interesting cases and new techniques in gastroenterological endoscopy. All papers include a high-quality video and are published with a Creative Commons CC-BY license. Endoscopy E-Videos qualify for HINARI discounts and waivers and eligibility is automatically checked during the submission process. We grant 100% waivers to articles whose corresponding authors are based in Group A countries and 50% waivers to those who are based in Group B countries as classified by Research4Life (see: https://www.research4life.org/access/eligibility/ ). This section has its own submission website at https://mc.manuscriptcentral.com/e-videos .
